# Impact of Coronary Microvascular Dysfunction on Patient-Reported Symptoms After PCI

**DOI:** 10.1016/j.jacadv.2026.102946

**Published:** 2026-07-13

**Authors:** Frédéric Bouisset, Kazumasa Ikeda, Takuya Mizukami, Daniel Munhoz, Jeroen Sonck, Koshiro Sakai, Hitoshi Matsuo, Hirohiko Ando, Brian Ko, Simone Biscaglia, Fernando Rivero, Antonio Maria Leone, Andy Yong, Javier Escaned, Damien Collison, Gianluca Campo, Liyew Desta, Tetsuya Amano, Toshiro Shinke, William F. Fearon, Ethan Korngold, Evald Høj Christiansen, Colin Berry, Divaka Perera, Bernard De Bruyne, Nils P. Johnson, Carlos Collet

**Affiliations:** a Department of Cardiology Toulouse University Hospital, France; b Department of Cardiology, Tokyo Medical University Hachioji Medical Center, Tokyo, Japan; c Division of Clinical Pharmacology, Department of Pharmacology, Showa University, Tokyo, Japan; d Cardiovascular Center Aalst, OLV Clinic, Aalst, Belgium; e Department of Medicine, Division of Cardiology, Showa University School of Medicine, Tokyo, Japan; f Department of Cardiovascular Medicine, Gifu Heart Center, Gifu, Japan; g Department of Cardiology, Aichi Medical University, Aichi, Japan; h Monash Cardiovascular Research Centre, Monash University and Monash Heart, Monash Health, Clayton, Victoria, Australia; i Cardiology Unit, Azienda Ospedaliera Universitaria di Ferrara, Ferrara, Italy; j Cardiac Department, Hospital Universitario de La Princesa, IIS-IP, Madrid, Spain; k Center of Excellence in Cardiovascular Sciences, Ospedale Isola Tiberina Gemelli Isola, Rome, Italy; l Catholic University of the Sacred Heart, Rome, Italy; m Concord Repatriation General Hospital, University of Sydney, New South Wales, Australia; n Instituto de Investigacion Sanitaria del Hospital Clinico San Carlos and Complutense University, Madrid, Spain; o School Cardiovascular and Metabolic Health, University of Glasgow, Glasgow, United Kingdom; p NHS Golden Jubilee Hospital, Clydebank, United Kingdom; q Department of Cardiology, Karolinska University Hospital, Solna, Stockholm, Sweden; r Division of Cardiovascular Medicine and Stanford Cardiovascular Institute, Stanford University School of Medicine and VA Palo Alto Health Care System, Palo Alto, California, USA; s Abbott Vascular; t Department of Cardiology, Aarhus University Hospital, Skejby, Aarhus, Denmark; u School of Cardiovascular Medicine and Sciences, St Thomas’ Hospital Campus, King's College London, London, United Kingdom; v Weatherhead PET Center, Division of Cardiology, Department of Medicine, McGovern Medical School at UTHealth and Memorial Hermann Hospital, Houston, Texas, USA; w Clinical Trial Center, Cardiovascular Research Foundation, New York, New York, USA

**Keywords:** coronary microvascular function, fractional flow reserve, microvascular resistance reserve, Per-cutaneous coronary intervention, pull back pressure gradient

## Abstract

**Background:**

Coronary microvascular dysfunction (CMD) has been proposed as a mechanism underlying residual angina after percutaneous coronary intervention (PCI).

**Objectives:**

The objective of the study was to investigate the impact of CMD on symptoms in patients undergoing PCI.

**Methods:**

Patients with hemodynamically significant coronary artery disease (CAD) (fractional flow reserve ≤0.80) were included. CAD was classified as focal or diffuse using the pull back pressure gradient (PPG) (diffuse CAD defined as PPG <0.62). CMD was defined as microvascular resistance reserve <3.0. The Seattle Angina Questionnaire (SAQ) was administered at baseline and 1 year.

**Results:**

Among 201 patients (mean age 68.5 ± 10.1 years; 71% male), CMD was present in 75 (37.3%), with no difference between focal and diffuse CAD (41% vs 34%; *P* = 0.35). At baseline, CMD was associated with more severe symptoms without reaching statistical significance (SAQ summary score 64.0 ± 25.3 vs 69.6 ± 21.0; *P* = 0.09). At 1 year, symptoms were similar between groups (SAQ summary score 87.6 ± 16.0 vs 89.4 ± 16.4; *P* = 0.47). A significant interaction between PPG and microvascular resistance reserve was observed for residual angina (*P* for interaction = 0.015); patients with focal CAD and concomitant CMD had the highest burden of residual symptoms.

**Conclusions:**

CMD is present in approximately one-third of patients undergoing PCI and occurs with similar frequency in focal and diffuse CAD. CMD alone was not associated with residual angina. However, its clinical relevance varied according to the epicardial disease pattern: in focal CAD, concomitant CMD was associated with less symptomatic improvement after PCI, whereas in diffuse CAD, residual symptoms appeared to be driven predominantly by persistent epicardial disease.

Percutaneous coronary intervention (PCI) effectively alleviates angina in patients with coronary artery disease (CAD).^1^ However, approximately 30% to 60% of patients continue to experience symptoms after a successful PCI.^1^, ^2^, ^3^, ^4^ Residual angina after PCI is commonly attributed to suboptimal epicardial revascularization—often due to diffuse disease—and the presence of coronary microvascular dysfunction (CMD). Although CMD is frequently cited as a potential contributor, clinical evidence supporting its role in residual angina remains limited.

Microvascular function can be assessed invasively using the microvascular resistance reserve (MRR).^5^ MRR is specific to the microvasculature and independent of epicardial disease, making it an ideal metric for patients with concomitant CAD.^3^^,^^4^^,^^6^, ^7^, ^8^ Epicardial disease can be further characterized based on the pattern of pressure loss (focal or diffuse). Previous reports have linked diffuse disease with CMD. The pull back pressure gradient (PPG) is a novel metric derived from pressure pull back curves, and quantifies diffuse and focal disease on a scale from 1 (focal) to 0 (diffuse).^9^ Diffuse CAD, as defined by the PPG, has been associated with residual angina after PCI.^10^

In this study, we sought to elucidate the mechanism underlying residual angina after PCI by comprehensively assessing the coronary circulation using PPG and MRR in patients with flow-limiting coronary stenosis planned for PCI.

## Methods

### Study design

This is a predefined subanalysis of the PPG Global Registry (NCT04789317). Briefly, PPG Global was a prospective, investigator-initiated, multicenter, international, and single-arm study. Study design and main results have already been published.^10^^,^^11^ Patients were eligible if aged 18 years and older with either chronic coronary syndrome or a nonculprit lesion during an acute coronary syndrome, at least 1 stenosis in a major epicardial vessel with distal fractional flow reserve (FFR) ≤0.80, and a candidate for revascularization by PCI. Patients were excluded during acute myocardial infarction, reduced left ventricular ejection fraction (<30%), poor renal function (estimated glomerular filtration rate <30 mL/min/1.73 m^2^), aorto-ostial target lesion, severe vessel tortuosity, and planned two-stent bifurcation PCI. Every participant signed informed consent, and every site received approval from its local institutional review board. An independent clinical events committee adjudicated adverse events blinded to the invasive physiological data. An external core laboratory centralized data collection and analyzed imaging and physiologic data (CoreAalst BV). This substudy includes patients with additional microvascular assessment using bolus thermodilution, which was encouraged but not mandatory in the study protocol. The study was sponsored by the Cardiovascular Research Institute Aalst with a grant from Abbott Vascular.

### Procedure

Coronary angiography of the target lesion was acquired in 2 views at least 30 degrees apart after injecting 100 to 200 μg of intracoronary nitroglycerin. A coronary wire equipped with a distal pressure sensor (PressureWire X; Abbott Vascular) was introduced into the target vessel after pressure equalization at the tip of the guiding catheter. The pressure wire was positioned in the distal coronary artery in a segment ≥2 mm and at least 15 mm beyond the most distal stenosis by visual estimation. Standardized physiologic assessment was mandated, including measurements of nonhyperemic pressure ratios, distal FFR, and a manual pull back during hyperemia. The pull back maneuver was performed manually at a constant speed for 20 to 30 seconds. When the pressure sensor reached the catheter tip, the pull back recording was stopped, and PPG was calculated onsite using CoroFlow software (v3.5.1; Coroventis Research AB). The calculation of the PPG involves the integration of 2 parameters derived from the pressure pull back curve, specifically the maximal pressure gradient over a 20% window of the pull back duration and the extent of functional disease over the complete pull back curve.^9^

### Microvascular function evaluation

Bolus thermodilution was performed at the physician’s discretion before and/or after PCI using three 3-mL saline injections at rest and during hyperemia to obtain mean transit time. Coronary flow reserve (CFR), index of microvascular resistance (IMR), corrected IMR (IMRcorr), and MRR were calculated using standard, previously validated formulas implemented in the CoroFlow software (see Supplementary Methods for detailed equations).

All angiographic and physiologic data underwent centralized, independent review at the CoreAalst core laboratory. Quantitative coronary angiography was performed via 2 views using 3-dimensional quantitative coronary angiography with CAAS 8.2 software (Pie Medical Imaging). Offline evaluation of physiology tracings was conducted using CoroFlow software (Coroventis Research AB). All tracings were reviewed by physicians experienced in physiology measurements.

### Patient-reported outcomes

The Seattle Angina Questionnaire (SAQ) of 7 items was administrated at baseline and repeated at the 12-month follow-up to assess symptoms.^12^ The SAQ score assesses 3 domains: angina frequency (AF), physical limitation, and quality of life (QOL). A score of 100 (maximum possible) indicates the best health status; freedom from angina implies a score of 100 in the AF domain.^13^

### Statistical analysis

Analyses were performed using R (version 4.3.1; R Foundation for Statistical Computing). Continuous variables were assessed for distribution and are presented as mean ± SD or median with IQR, as appropriate. Categorical variables are presented as counts and percentages. Between-group comparisons were performed using the Student’s *t*-test or Mann-Whitney U test for continuous variables, and the chi-square test or Fisher exact test for categorical variables, as appropriate. Correlations between physiological indices were assessed using Pearson or Spearman correlation coefficients according to variable distribution.

The median PPG value of 0.62 was used for the main analysis to categorize CAD pattern as focal vs diffuse disease. CMD was defined as MRR <3.0. Patient-reported outcomes were compared between patients with and without CMD in the overall cohort and according to CAD pattern. Logistic regression models were used to evaluate the association between physiological indices and residual symptoms at 12 months, defined as SAQ domain or summary scores <100. Models were adjusted for age and sex. Interaction between CAD pattern and CMD status was tested by including an interaction term in the model. Effect estimates are reported with 95% CIs. All tests were 2-sided, and *P* < 0.05 was considered statistically significant.

## Results

**Figure 1:**
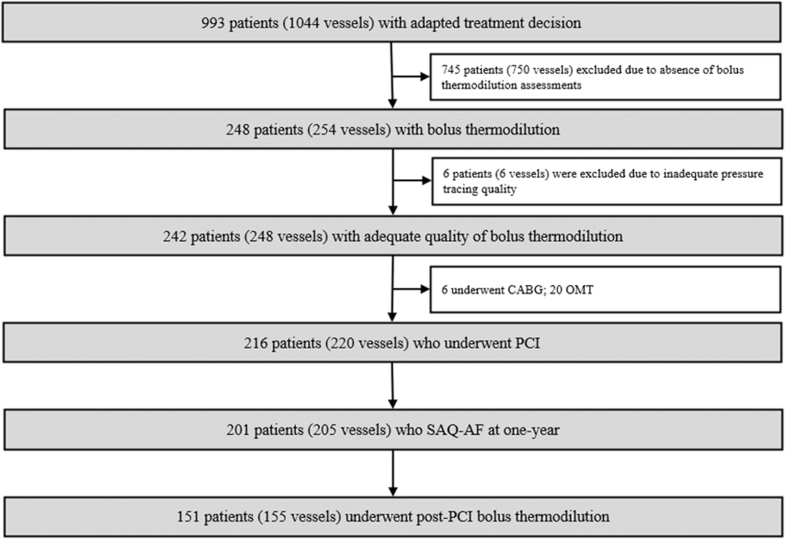
Study Flowchart CABG = coronary artery bypass grafting; OMT = optimal medical therapy; PCI = percutaneous coronary intervention; SAQ-AF = Seattle Angina Questionnaire Angina Frequency.

Overall, 248 patients had a complete evaluation of coronary circulation using FFR, PPG, CFR, IMR, and MRR. Six patients were excluded due to poor temperature tracing quality. Therefore, 242 patients with complete epicardial and microvascular assessments were included (Figure 1). Supplemental Table 1 shows a comparison between patients undergoing CMD assessment and those who did not. Among the 242 patients included, 216 were treated with PCI (6 underwent coronary artery bypass grafting and 20 received medical therapy alone). Of the 216 patients treated with PCI, 201 completed the SAQ-7 questionnaire at 1 year of follow-up.

The mean age was 68.5 ± 10.1, 71.1% of patients were males, and 32.3% had diabetes. Patients with CMD were older, more frequently females, and had more arterial hypertension (Table 1). The target vessel was the left anterior descending artery in 79.0% of cases. The mean FFR and PPG were 0.70 ± 0.10 and 0.64 ± 0.16. Procedural characteristics are shown in Table 2.

**Table 1:** Baseline Clinical Characteristics

	All Patients (N = 201)	CMD (n = 75)	Normal Microvascular Function (n = 126)	*P* Value
Age, y, mean ± SD	68.5 ± 10.1	70.8 ± 9.6	67.2 ± 10.1	0.015
Male, n (%)	143 (71.1)	47 (62.7)	96 (76.2)	0.059
BMI, mean ± SD	26.1 ± 4.0	25.7 ± 4.1	26.4 ± 3.9	0.185
Dyslipidemia, n (%)	147 (73.1)	55 (73.3)	92 (73.0)	1.000
Hypertension, n (%)	148 (73.6)	64 (85.3)	84 (66.7)	0.006
Diabetes, n (%)	65 (32.3)	30 (40.0)	35 (27.8)	0.102
Smoking, n (%)	33 (16.4)	11 (14.7)	22 (17.5)	0.749
Prior PCI in nontarget vessel, n (%)	53 (26.4)	19 (25.3)	34 (27.0)	0.421
Prior PCI in target vessel, n (%)	22 (10.9)	6 (8.0)	16 (12.7)	0.261
Prior MI, n (%)	39 (19.4)	13 (17.3)	26 (20.6)	0.698
PAD, n (%)	7 (3.5)	2 (2.7)	5 (4.0)	0.929
Clinical presentation				0.222
Non-STEMI	12 (6.0)	7 (9.3)	5 (4.0)	
Unstable angina	10 (5.0)	6 (8.0)	4 (3.2)	
Chronic coronary syndrome				0.470
Asymptomatic	20 (10.0)	7 (9.3)	13 (10.3)	
Silent ischemia	26 (12.9)	8 (10.7)	18 (14.3)	
Stable angina CCS I	60 (29.9)	16 (21.3)	44 (34.9)	
Stable angina CCS II	54 (26.9)	24 (32.0)	30 (23.8)	
Stable angina CCS III	15 (7.5)	5 (6.7)	10 (7.9)	
Stable angina CCS IV	4 (2.0)	2 (2.7)	2 (1.6)	
Creatinine clearance, mean ± SD	72.5 ± 24.8	72.5 ± 27.9	72.5 ± 22.9	0.992
LVEF, mean ± SD	58.7 ± 10.5	59.0 ± 11.9	58.5 ± 9.5	0.768

Per patient analysis.

BMI = body mass index; CCS = Canadian Cardiovascular Society; CMD = coronary microvascular dysfunction; non-CMD = noncoronary microvascular dysfunction; LVEF = left ventricular ejection fraction; MI = myocardial infarction; Non-STEMI = non-ST elevation myocardial infarction; PAD = peripheral arterial disease; PCI = per-cutaneous intervention.

**Table 2:** Procedural Characteristics

	All Vessels (N = 205)	CMD (n = 78)	Normal Microvascular Function (n = 127)	*P* Value
Target vessel, n (%)				0.069
LAD	162 (79.0)	56 (71.8)	106 (83.5)	
LCX	16 (7.8)	10 (12.8)	6 (4.7)	
RCA	27 (13.2)	12 (15.4)	15 (11.8)	
Pre-PCI				
Pre-PCI DS (%), mean ± SD	50.1 ± 13.4	52.5 ± 11.3	48.6 ± 14.4	0.045
Pre-PCI lesion length, mean ± SD	19.8 ± 12.1	22.4 ± 13.9	18.2 ± 10.6	0.015
FFR pre-PCI	0.70 ± 0.10	0.71 ± 0.08	0.69 ± 0.11	0.126
PPG pre-PCI	0.64 ± 0.16	0.65 ± 0.14	0.62 ± 0.16	0.192
CAD pattern				
Focal, n (%)	104 (50.7)	44 (56.4)	60 (47.2)	0.258
CFR pre-PCI	2.34 ± 1.18	1.40 ± 0.40	2.92 ± 1.12	<0.001
IMR pre-PCI	20.9 ± 12.6	22.0 ± 12.4	20.2 ± 12.7	0.316
IMR_corrected_ pre-PCI	17.9 ± 10.8	19.5 ± 11.1	16.9 ± 10.6	0.101
MRR pre-PCI	3.80 ± 1.87	2.17 ± 0.55	4.80 ± 1.67	<0.001
Total stent length, mean ± SD	30.1 ± 15.4	30.6 ± 16.1	29.7 ± 14.9	0.684
Mean stent diameter, mean ± SD	3.04 ± 0.42	2.94 ± 0.41	3.10 ± 0.42	0.011
Number of stent, mean ± SD	1.20 ± 0.47	1.24 ± 0.54	1.17 ± 0.41	0.253
Post-PCI				
Post-PCI DS, mean ± SD	0.22 ± 16.97	2.14 ± 17.36	−0.93 ± 16.70	0.221
Post-PCI lesion length, mean ± SD	28.8 ± 13.3	29.1 ± 14.1	28.6 ± 12.7	0.772
FFR post-PCI	0.87 ± 0.06	0.87 ± 0.06	0.87 ± 0.06	0.988
Delta FFR	0.18 ± 0.12	0.16 ± 0.10	0.19 ± 0.13	0.190
CFR post-PCI	3.10 ± 1.87	2.37 ± 1.22	3.54 ± 2.04	<0.001
IMR post-PCI	18.2 ± 11.9	17.7 ± 11.3	18.5 ± 12.2	0.690
IMR_corrected_ post-PCI	17.7 ± 11.7	17.5 ± 11.3	17.7 ± 12.0	0.915
MRR post-PCI	4.18 ± 2.55	3.36 ± 2.40	4.68 ± 2.52	0.002
Delta CFR	0.78 ± 1.87	0.98 ± 1.22	0.66 ± 2.16	0.308
Delta IMR	−2.97 ± 12.3	−4.50 ± 11.9	−2.05 ± 12.5	0.234
Delta MRR	0.36 ± 2.69	1.15 ± 2.39	−0.11 ± 2.76	0.004

Per vessel analysis.

CAD = coronary artery disease; CFR = coronary flow reserve; FFR = fractional flow reserve; IMR = index of microvascular resistance; IMRcorr = index of microvascular resistance corrected; LAD = left anterior descending; LCX = left circumflex; MRR = microvascular resistance reserve; PPG = pull back pressure gradient; RCA = right coronary artery; other abbreviations as in Table 1.

### Baseline epicardial and microvascular assessments

**Figure 2:**
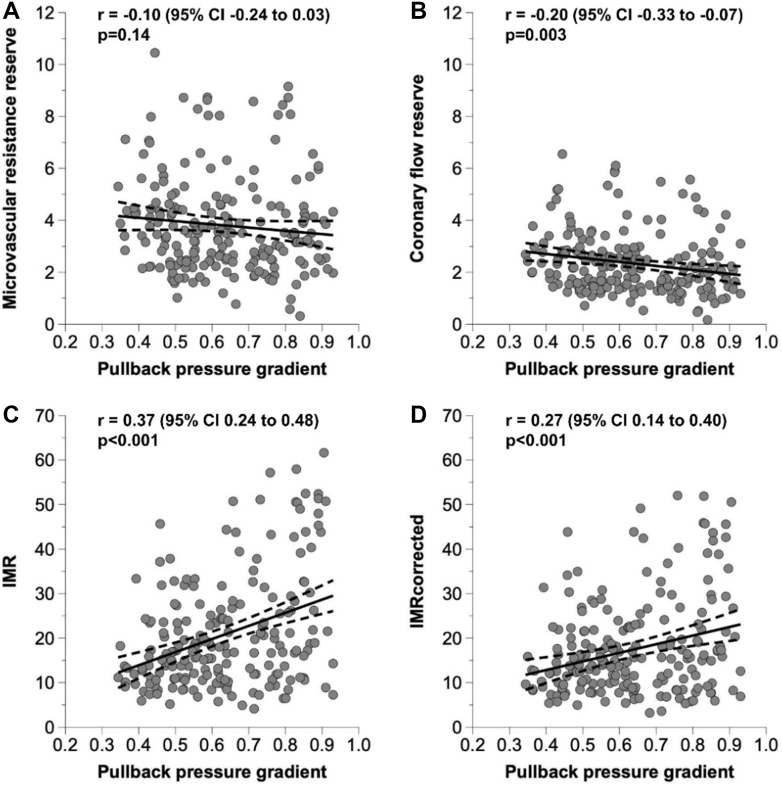
Correlations Between Pull Back Pressure Gradient and Microvascular Resistance Reserve, Coronary Flow Reserve, Index of Microvascular Resistance, and Index of Microvascular Resistance Corrected Panels A–D show the correlations between PPG and (A) MRR, (B) CFR, (C) IMR, and (D) IMR_corr_, respectively. No significant correlation was observed between PPG and MRR. In contrast, PPG showed a significant but weakly negative correlation with CFR and significant but weakly positive correlations with both IMR and IMR_corr_.

The mean CFR, IMR, and MRR were 2.34 ± 1.18, 20.9 ± 12.6, and 3.80 ± 1.87, respectively. CFR and IMR were significantly but weakly correlated with PPG (CFR r = −0.20 with 95% CI: −0.33 to −0.07; *P* = 0.003, and IMR 0.37 with 95% CI: 0.24-0.48; *P* < 0.001). There was no correlation between MRR and PPG (r = −0.1; 95% CI: −0.24 to 0.03; *P* = 0.14) (Figure 2).

### CMD prevalence

**Figure 3:**
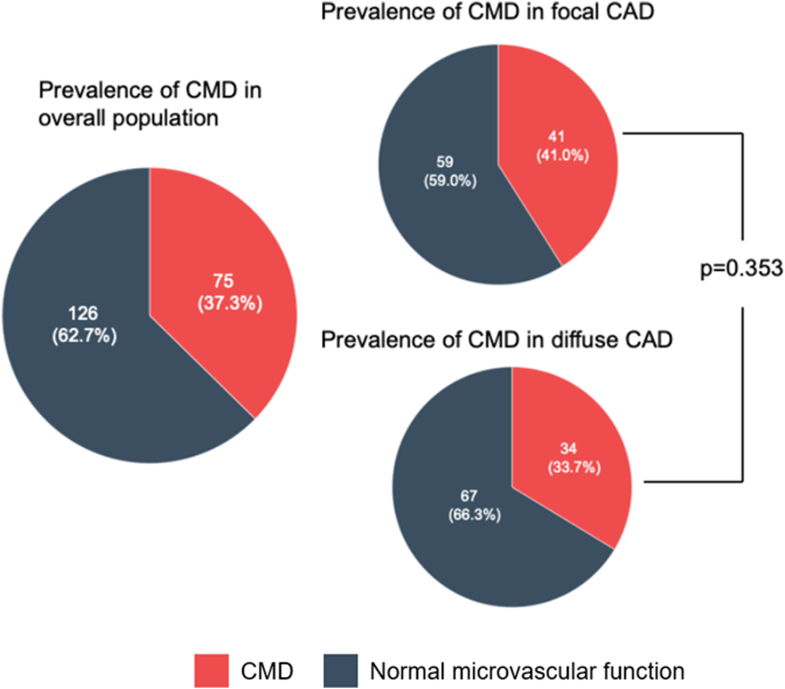
Proportion of CMD Patients in the Entire Cohort and Stratified by CAD Pattern The large pie chart illustrates the overall prevalence of CMD, with 37.3% of patients exhibiting CMD (red) and 62.7% without CMD (dark blue). The smaller pie charts show CMD prevalence by CAD phenotype: 41% in patients with focal CAD and 33.7% in those with diffuse CAD (red). The difference in CMD prevalence between focal and diffuse CAD was not statistically significant (*P* = 0.35). CAD = coronary artery disease; CMD = coronary microvascular dysfunction.

CMD (MRR <3.0) was present in 37.3% of patients and showed similar prevalence between focal and diffuse CAD (41% vs 33.74%; *P* = 0.35) (Figure 3). However, when alternative definitions of CMD based on CFR and/or IMR were applied, CMD was more frequently observed in patients with focal CAD (Supplemental Figure 1).

### Post-PCI epicardial and microvascular assessments

The mean post-PCI FFR was similar between patients with and without CMD (0.87 ± 0.06 vs 0.87 ± 0.06; *P* = 0.98). Post-PCI FFR did not differ between CMD and non-CMD patients within either focal CAD (0.89 ± 0.06 vs 0.90 ± 0.06; *P* = 0.23) or diffuse CAD (0.86 ± 0.05 vs 0.85 ± 0.06; *P* = 0.52) (Supplemental Table 2).

Post-PCI microvascular assessments were available in 151 patients (155 vessels): the mean CFR was 3.10 ± 1.87, IMR 18.2 ± 11.9, IMR_corr_ 17.7 ± 11.7 and MRR 4.18 ± 2.55. Compared to pre-PCI, there were significant improvements in CFR (2.34 ± 1.18 vs 3.10 ± 1.87; *P* < 0.001) and a reduction in IMR (20.1 ± 12.6 vs 18.2 ± 11.9; *P* = 0.04). No significant changes were observed in IMRcorr (17.9 ± 10.8 vs 17.7 ± 11.7; *P* = 0.84) or MRR (3.80 ± 1.87 vs 4.18 ± 2.55; *P* = 0.099) (Supplemental Figure 2). Supplemental Table 3 shows a comparison between patients who underwent post-PCI microvascular assessment and those who did not.

### Patient-reported outcomes

**Figure 4:**
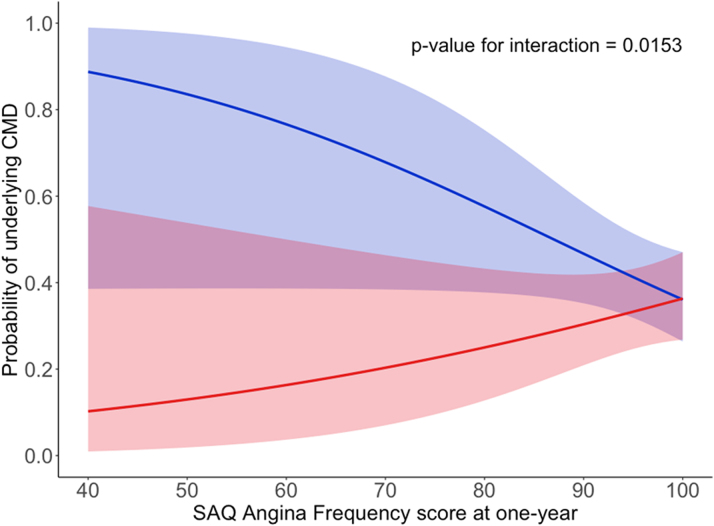
Probability of Underlying CMD Based on 12-Month Functional Status, Stratified by Coronary Artery Disease Pattern This figure illustrates the modeled probability of CMD in relation to residual angina, as measured by the SAQ-AF score. Among patients with focal CAD (blue), lower SAQ-AF scores—reflecting more frequent residual angina—were associated with a higher probability of CMD. In contrast, CMD probability remained relatively stable across the range of SAQ-AF scores in patients with diffuse CAD (red). A significant interaction was observed between CAD pattern and SAQ score in predicting CMD (*P* for interaction = 0.015). Shaded areas indicate 95% CIs. Abbreviations as in Figures 1 and 3.

Patients with CMD (MRR <3.0) had a higher frequency of angina, greater physical limitations, and lower SAQ summary scores compared to patients with normal microvascular function (Table 3). After PCI, there were no significant differences in residual angina between patients with and without CMD (SAQ-AF 94.80 ± 10.83 vs 95.24 ± 11.50, *P* = 0.79). Interestingly, among patients with focal CAD, those with CMD reported more residual angina (SAQ-AF 92.93 ± 12.89 vs 96.95 ± 6.50; *P* = 0.043) and lower QOL (SAQ-QOL 78.66 ± 25.04 vs 88.35 ± 17.35; *P* = 0.024). In contrast, no significant differences in symptoms were observed between CMD and non-CMD patients with diffuse disease after revascularization (Table 3). A significant interaction between PPG and CMD was observed for residual angina after PCI (*P* for interaction = 0.015) (Figure 4).

**Table 3:** Baseline and 12 Months Patient-Reported Outcome According to CMD Status

SAQ	All Patients (N = 201)	CMD (n = 75)	Normal Microvascular Function (n = 126)	*P* Value
Entire Population				
Baseline				
Physical limitation	74.58 ± 29.17	70.21 ± 32.92	77.13 ± 26.55	0.107
Angina frequency	77.76 ± 22.03	74.27 ± 22.97	79.84 ± 21.28	0.083
Quality of life	51.18 ± 29.57	49.00 ± 31.42	52.48 ± 28.46	0.421
Summary score	67.56 ± 22.82	64.00 ± 25.33	69.64 ± 21.04	0.093
1-year				
Physical limitation	87.66 ± 23.61	86.32 ± 22.19	88.41 ± 24.43	0.563
Angina frequency	95.07 ± 11.23	94.80 ± 10.83	95.24 ± 11.50	0.790
Quality of life	84.08 ± 22.32	83.33 ± 22.16	84.52 ± 22.50	0.716
Summary score	88.74 ± 16.22	87.59 ± 16.04	89.39 ± 16.36	0.469
Delta				
Physical limitation	13.26 ± 30.47	12.82 ± 31.21	13.51 ± 30.18	0.882
Angina frequency	17.31 ± 20.34	20.53 ± 22.11	15.40 ± 19.04	0.083
Quality of life	32.90 ± 32.51	34.33 ± 33.58	32.04 ± 31.96	0.630
Summary score	20.29 ± 23.53	22.13 ± 24.19	19.27 ± 23.19	0.431

	**All Patients (N = 100)**	**CMD (n = 41)**	**Normal Microvascular Function (n = 59)**	

Focal CAD				
Baseline				
Physical limitation	69.63 ± 30.14	63.82 ± 33.20	73.66 ± 27.39	0.109
Angina frequency	75.00 ± 21.39	70.24 ± 21.27	78.31 ± 21.02	0.064
Quality of life	47.12 ± 28.53	45.73 ± 30.82	48.09 ± 27.05	0.686
Summary score	63.92 ± 22.55	59.93 ± 25.07	66.69 ± 20.39	0.142
1-year				
Physical limitation	86.90 ± 23.47	82.13 ± 24.94	89.97 ± 22.14	0.108
Angina frequency	95.30 ± 9.79	92.93 ± 12.89	96.95 ± 6.50	0.043
Quality of life	84.38 ± 21.27	78.66 ± 25.04	88.35 ± 17.35	0.024
Summary score	88.78 ± 15.44	84.15 ± 18.47	91.76 ± 12.40	0.017
Delta				
Physical limitation	17.53 ± 32.30	16.34 ± 34.77	18.32 ± 30.87	0.771
Angina frequency	20.30 ± 21.15	22.68 ± 21.22	18.64 ± 21.13	0.350
Quality of life	37.25 ± 31.08	32.93 ± 35.00	40.25 ± 27.96	0.248
Summary score	24.46 ± 22.81	23.52 ± 25.45	25.07 ± 21.15	0.745

	**All Patients (N = 101)**	**CMD (n = 34)**	**Normal Microvascular Function (n = 67)**	***P* Value**

Diffuse CAD				
Baseline				
Physical limitation	79.63 ± 27.38	78.39 ± 31.19	80.24 ± 25.57	0.755
Angina frequency	80.50 ± 22.42	79.12 ± 24.29	81.19 ± 21.57	0.662
Quality of life	55.20 ± 30.17	52.94 ± 32.14	56.34 ± 29.30	0.595
Summary score	71.27 ± 22.59	69.20 ± 25.07	72.28 ± 21.42	0.530
One-year				
Physical limitation	88.48 ± 23.86	91.81 ± 16.83	86.88 ± 26.58	0.363
Angina frequency	94.85 ± 12.54	97.06 ± 7.19	93.73 ± 14.44	0.209
Quality of life	83.79 ± 23.42	88.97 ± 16.79	81.16 ± 25.87	0.114
Summary score	88.71 ± 17.13	92.10 ± 10.88	87.07 ± 19.31	0.196
Delta				
Physical limitation	8.43 ± 27.67	8.04 ± 25.43	8.63 ± 28.92	0.927
Angina frequency	14.36 ± 19.15	17.94 ± 23.20	12.54 ± 16.64	0.182
Quality of life	28.59 ± 33.46	36.03 ± 32.24	24.81 ± 33.67	0.112
Summary score	15.65 ± 23.57	20.24 ± 22.69	13.47 ± 23.85	0.213

SAQ = Seattle Angina Questionnaire; other abbreviations as in Tables 1 and 2.

When CMD was defined using alternative criteria—such as low CFR alone, high IMR (either corrected or uncorrected), or a combination of low CFR and high IMR—no significant association was observed between microvascular status and patient-reported symptom at 12-month follow-up (Supplemental Table 4).

## Discussion

This study highlights several key findings about the complex interplay between epicardial and microvascular disease (Central Illustration). First, CMD was present in approximately one-third of patients with hemodynamically significant epicardial CAD, with a similar prevalence observed in both focal and diffuse patterns of disease. Second, CMD was associated with more severe anginal symptoms before PCI. After PCI, residual symptoms and QOL were similar between patients with CMD and those with normal microvascular function. Third, we found a significant interaction between PPG and MRR, indicating that in patients with focal CAD, the presence of CMD was linked to a greater burden of residual angina following revascularization.

**Central Illustration:**
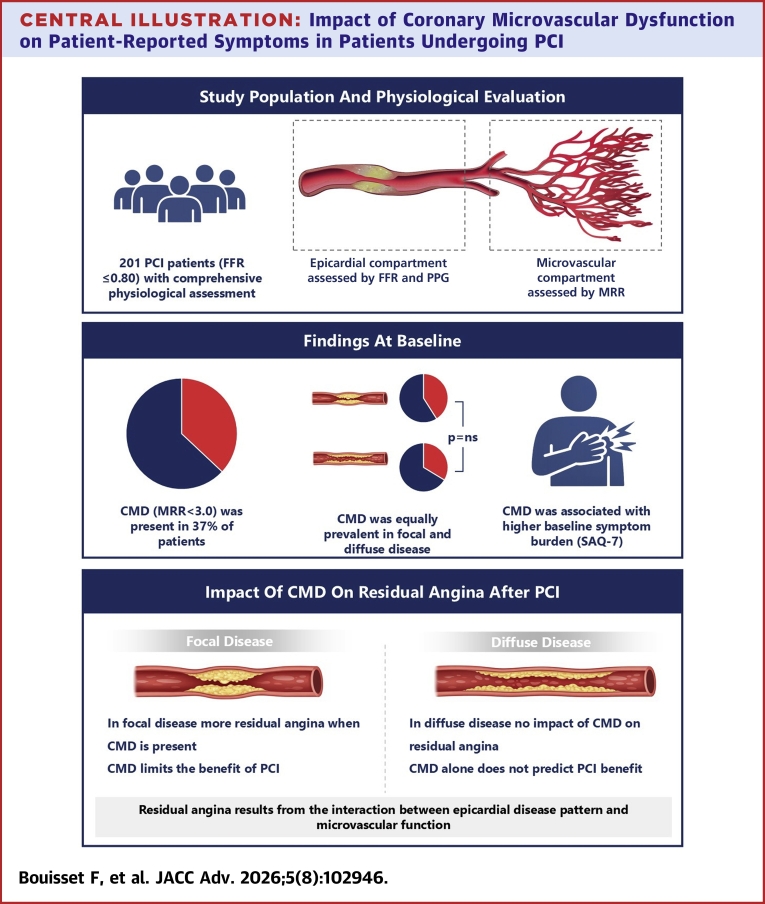
Impact of Coronary Microvascular Dysfunction on Patient-Reported Symptoms in Patients Undergoing PCI Coronary microvascular dysfunction (CMD), defined as MRR <3.0, was present in approximately one-third of patients undergoing PCI with comprehensive physiological assessment and was similarly prevalent in focal and diffuse epicardial disease. At baseline, CMD was associated with greater symptom burden as assessed by SAQ-7. However, CMD alone did not uniformly predict residual angina after PCI. In focal coronary artery disease, CMD was associated with more residual angina, suggesting that microvascular dysfunction may limit the symptomatic benefit of successful epicardial revascularization. In contrast, in diffuse coronary artery disease, residual symptoms appeared to be mainly driven by persistent epicardial disease rather than CMD. FFR = fractional flow reserve; MRR = microvascular resistance reserve; PCI = percutaneous coronary intervention; PPG = pull back pressure gradient; SAQ-AF = Seattle Angina Questionnaire Angina Frequency.

### Prevalence of CMD in patients with epicardial coronary artery disease

The prevalence of CMD has primarily been studied in patients presenting with angina or ischemia without obstructive CAD (angina with no obstructive coronary artery disease or ischemia with no obstructive coronary artery disease). According to a recent meta-analysis including more than 14,000 patients, CMD was found to be present in 41% of patients without obstructive CAD.^14^ This finding also aligns with the observation that nearly half of patients undergoing coronary angiography for angina do not have significant obstructive coronary disease.^15^

Earlier, smaller studies have suggested that microvascular dysfunction may also be present in patients with significant epicardial CAD, such as the work by Qian et al^16^, who reported impaired microvascular function in a subset of patients with angiographically significant coronary disease using Doppler-based methods. However, these studies were limited by sample size and heterogeneous methodologies.

To the best of our knowledge, no previous study has examined the prevalence of underlying CMD in a large population with significant epicardial CAD. The present study reveals that CMD is common, occurring in 1 of 3 patients with flow-limiting epicardial CAD, and is equally prevalent in focal and diffuse CAD.

From a methodological perspective, it is important to note that MRR was originally developed using continuous thermodilution, whereas in the present study it was derived from bolus thermodilution. This methodological difference is relevant, as bolus-derived measurements are known to exhibit greater variability than those obtained with continuous thermodilution. Recent studies have nevertheless demonstrated the feasibility and clinical relevance of bolus-derived MRR, including a direct comparison between continuous and bolus techniques by Gallinoro et al^17^^,^^18^ and its application in contemporary clinical cohorts by de Vos et al^19^

### Impact of underlying CMD on residual angina

Persistent post-PCI angina is a challenge in clinical practice. In 2015, Li et al^20^ reported a series of 39 patients with post-PCI angina and no significant epicardial disease. Their findings revealed that, compared to 12 control patients, those with persistent angina exhibited higher IMR and lower CFR values, suggesting that CMD may underlie persistent symptoms. More recently, Cui et al^21^ reported in a cohort of 102 patients with post-PCI angina that CMD, detected via myocardial perfusion imaging, was present in approximately 50% of cases.

In the present study, CMD was similarly prevalent in focal and diffuse CAD, yet its symptomatic relevance revascularization differed according to the epicardial disease pattern, as demonstrated by the significant interaction between PPG and MRR on SAQ outcomes (Figure 4). Importantly, post-PCI FFR values were comparable between CMD and non-CMD patients within both focal CAD (0.89 ± 0.06 vs 0.90 ± 0.06) and diffuse CAD (0.86 ± 0.05 vs 0.85 ± 0.06) (Supplemental Table 2), indicating that the observed differences in symptom relief are not explained by residual epicardial disease. These findings support the hypothesis that the clinical expression of CMD depends on the epicardial context in which it occurs. In diffuse CAD, PCI typically yields only modest improvements in flow, and a substantial proportion of patients remain symptomatic, likely due to residual epicardial disease and incomplete functional revascularization. In contrast, focal lesions respond to PCI with a pronounced increase in flow, often resulting in optimal post-PCI FFR and substantial angina relief.^9^ In this setting, persistent symptoms despite successful PCI may unmask the presence of concomitant microvascular dysfunction. Thus, while residual angina in diffuse CAD may reflect either persistent epicardial resistance or CMD, in focal CAD it appears more closely linked to underlying microvascular disease.

### Study limitations

This study has several limitations. First, although the original PPG Global Registry enrolled more than 1,000 patients, only a subset underwent comprehensive microvascular assessment, resulting in a relatively modest sample size for the present analysis. In addition, the decision to perform microvascular measurements was left to operator discretion, which may introduce selection bias. Consequently, the present findings should be interpreted as hypothesis-generating.

Symptoms were assessed at only 2 time points using the SAQ, an instrument that has not been specifically validated in patients with microvascular disease. The absence of early and serial post-PCI assessments (eg, at 1-3 months) limits our ability to capture the temporal trajectory of symptom relief and may obscure transient differences between groups. Future studies employing tools, such as digital symptom tracking platforms (eg, the ORBITA app), may provide deeper insights into symptom dynamics.

From a technical standpoint, MRR was derived using bolus thermodilution, which is inherently less precise than continuous thermodilution. However, bolus thermodilution offers practical advantages: it can be performed rapidly during PCI using the same pressure guidewire as FFR, requires no additional equipment or cost, and is therefore well suited for large, real-world studies. It should also be acknowledged that bolus thermodilution assesses only endothelium-independent CMD. Endothelium-dependent dysfunction, which may be detected by acetylcholine testing, was not evaluated in this study and is therefore not captured in the present analysis.

## Conclusions

CMD was present in approximately one-third of patients with hemodynamically significant epicardial CAD and was equally prevalent in both focal and diffuse disease. Although CMD was associated with more severe anginal symptoms at baseline, its clinical relevance after PCI depended on the epicardial disease pattern. In patients with focal CAD, concomitant CMD was associated with less symptomatic improvement despite successful epicardial revascularization, whereas in patients with diffuse CAD, CMD was not associated with residual angina after revascularization.

Among the various physiological indices, MRR provided a refined characterization of microvascular function and contributed to identifying patients in whom CMD may limit symptomatic benefit from PCI. These findings underscore that residual angina after PCI cannot be explained by a single physiological abnormality in isolation, but rather reflect the interplay between epicardial and microvascular disease. Incorporating a comprehensive epicardial assessment with PPG together with microvascular evaluation may improve patient stratification and help tailor management strategies in patients undergoing PCI. Future prospective, adequately powered studies are warranted to validate these observations and to determine whether targeting CMD, beyond optimal treatment of epicardial stenosis, can enhance symptom relief and improve long-term outcomes after revascularization.

Perspectives **COMPETENCY IN MEDICAL KNOWLEDGE:** In patients undergoing PCI for hemodynamically significant coronary artery disease, persistent symptoms after successful revascularization may reflect the combined contribution of epicardial disease pattern and coronary microvascular dysfunction (CMD). Integrating physiological assessment of epicardial disease (PPG) and microvascular function (MRR) may help identify patients in whom CMD is likely to limit symptomatic improvement despite optimal epicardial treatment.

**TRANSLATIONAL OUTLOOK:** Prospective studies are needed to determine whether integrating epicardial disease pattern and coronary microvascular function into PCI decision-making improves patient selection, enables targeted adjunctive therapies, and enhances symptom relief and clinical outcomes after revascularization.

## Funding support and author disclosures

The PPG Global Registry was sponsored by the Cardiovascular Research Institute Aalst with a grant from 10.13039/100011949Abbott Vascular. Dr Bouisset reports consultancy fees from Abbott, Boston Scientific, Shockwave, and GE healthcare. Dr Mizukami reports receiving research grants from 10.13039/100016242Boston Scientific; and speaker fees from Abbott Vascular, CathWorks, and Boston Scientific. Dr Munhoz reports a research grant provided by the CardioPaTh PhD programme; and speaker fees from Abbott Vascular. Dr Sonck has patents and pending patents on diagnostic methods for coronary artery disease. Dr Matsuo has received consulting fees from Kaneka and Zeon; and speaker’s fees from Abbott Medical Japan, Boston Scientific, Philips, and Amgen. Dr Ko has received consulting fees from Canon Medical, Abbott, and Medtronic. Dr Biscaglia received research grants provided by Sahajanand Medical Technologies (SMT), Medis Medical Imaging, Eukon S.r.l., Siemens Healthineers, General Electric (GE) Healthcare, and Insight Lifetech. Dr Leone reports receiving consultancy fees from Abbott and honoraria for sponsored symposiums from Abbott, Medtronic, and Abiomed. Dr Escaned is supported by the Intensification of Research Activity project INT22/00088 from the Spanish Instituto de Salud Carlos III; and received speaker and advisory board member fees from Abbott and Philips. Dr Collison has received consulting fees from Abbott. Dr Amano reports receiving lecture fees from Astellas Pharma, Astra Zeneca, Bayer, Daiichi Sankyo, and Bristol-Myers Squibb. Dr Fearon receives institutional research support from Abbott, CathWorks, and Medtronic; has consulting relationships with ShockWave, and has stock options from HeartFlow. Dr Korngold is an employee of Abbott Vascular. Dr Christiansen has received consulting fees from Abbott Medical Denmark A/S. Dr Yong has received minor honoraria from Abbott Vascular, and research grants from 10.13039/100011949Abbott Vascular and 10.13039/100004320Philips. Dr Berry receives research funding from the British Heart Foundation (RE/18/6134217, BHF/FS/17/26/32744, PG/19/28/34310); and is employed by the University of Glasgow which holds consultancy and research agreements for his work with Abbott Vascular, AstraZeneca, Boehringer Ingelheim, Coroventis Research, GlaxoSmithKline (GSK), HeartFlow, Menarini, Novartis, Servier, Siemens Healthcare, and Valo Health. Dr Perera has received research grant support from 10.13039/100011949Abbott Vascular, 10.13039/100020588HeartFlow, and 10.13039/100004320Philips. Dr Bruyne reports receiving consultancy fees from Boston Scientific and Abbott; and research grants from Coroventis Research, Pie Medical Imaging, CathWorks, Boston Scientific, Siemens, HeartFlow, and Abbott Vascular. Dr Johnson received internal funding from the Weatherhead PET Center for Preventing and Reversing Atherosclerosis; has received significant institutional research support from St. Jude Medical (CONTRAST, NCT02184117) and Philips Volcano (DEFINE-FLOW, NCT02328820) for other studies using intracoronary pressure and flow sensors; has an institutional licensing agreement with Boston Scientific for the smart-minimum FFR algorithm (now commercialized under 510(k) K191008); and has patents pending on diagnostic methods for quantifying aortic stenosis and TAVI physiolog, and on methods to correct pressure tracings from fluid-filled catheters. Dr Collet reports receiving research grants from Biosensors, Coroventis Research, Medis Medical Imaging, Pie Medical Imaging, CathWorks, Boston Scientific, Siemens, HeartFlow, and Abbott Vascular; and consultancy fees from HeartFlow, OpSens Medical, Abbott Vascular, and Philips Volcano; and has patents pending on diagnostic methods for coronary artery disease. All other authors have reported that they have no relationships relevant to the contents of this paper to disclose.
