# Supplementary material for: Impact of Coronary Microvascular Dysfunction on Patient-Reported Symptoms After PCI
Source: JACC Adv. 2026 Jul 13;5(8):102946. doi: 10.1016/j.jacadv.2026.102946 (PMC13382258; doi:10.1016/j.jacadv.2026.102946)
Supplement: Supplemental Material [file mmc1.pdf]

# Supplementary Materials

# Impact of Coronary Microvascular Dysfunction on Patient-Reported Symptoms After PCI

*Bouisset et al*

**Address for correspondence:**
Carlos Collet, MD, PhD.
Cardiovascular Research Foundation
1700 Broadway, New York, NY 10019, United States of America
Phone: +1 646-434-4500
E-mail: carloscollet@gmail.com
Twitter @ColletCarlos

## Table of Content

Calculation of Microvascular Indices ........ 3

Supplemental Figures ........ 4

Supplemental Figure S1. Prevalence of CMD based on different CMD definitions ........ 4

Supplemental Figure S2. Microvascular indexes before and after PCI. ........ 5

Supplementary Tables ........ 6

Supplementary Table S1. Comparison of clinical characteristics between patients with and without microvascular assessment. ........ 6

Supplemental Table S2: Post-PCI FFR according to epicardial pattern and CMD status ........ 7

Supplemental Table S3: Comparison of baseline characteristics in patients with versus without post-PCI microvascular measurements ........ 8

Supplemental Table S4: Physiological predictors of residual symptoms and impaired quality of life at 12 months. ........ 9

References ........ 10

**Calculation of Microvascular Indices**

Bolus thermodilution was performed at the physician's discretion before and/or after PCI. (1) Three 3 mL boluses of saline were administered during resting and hyperemic conditions to determine the mean transit time (Tmn) and derive Coronary Flow Reserve (CFR), Index of Microvascular Resistance (IMR), and MRR. Microvascular function was evaluated using CFR, IMR, IMR corrected ($IMR_{corr}$), and MRR. IMR was determined using the standard calculation (1).

$$IMR = P_{d,\ hyp} \text{ x } T_{mn, hyp}$$

where $Pd_{hyp}$ equals the pressure measured distally by the wire in the coronary artery and $Tmn_{hyp}$ represents the mean transit time during hyperemia. IMR corrected ($IMR_{corr}$) used the calculation described by Young (2).

$$IMR_{corr} = P_{a,\ hyp} \times T_{mn,\ hyp} \times ([1.35 \times P_{d,\ hyp}/P_{a,\ hyp}] - 0.32)$$

where $Pa_{hyp}$ equals the aortic pressure during hyperemia. CFR was determined using the standard calculation (1).

$$CFR = T_{mn,\ rest} / T_{mn,\ hyp}$$

where $Tmn_{rest}$ represents the mean transit time at rest. MRR was calculated offline as previously described(3) using the following equation:

$$MRR = (CFR/FFR) \text{ x } (P_{a,\ rest} / P_{a,\ hyp})$$

where $P_{a,\ rest}$ denotes aortic pressure at rest and $P_{a,\ hyp}$ aortic pressure during hyperemia.

## Supplemental Figures

**Supplemental Figure S1. Prevalence of CMD based on different CMD definitions**

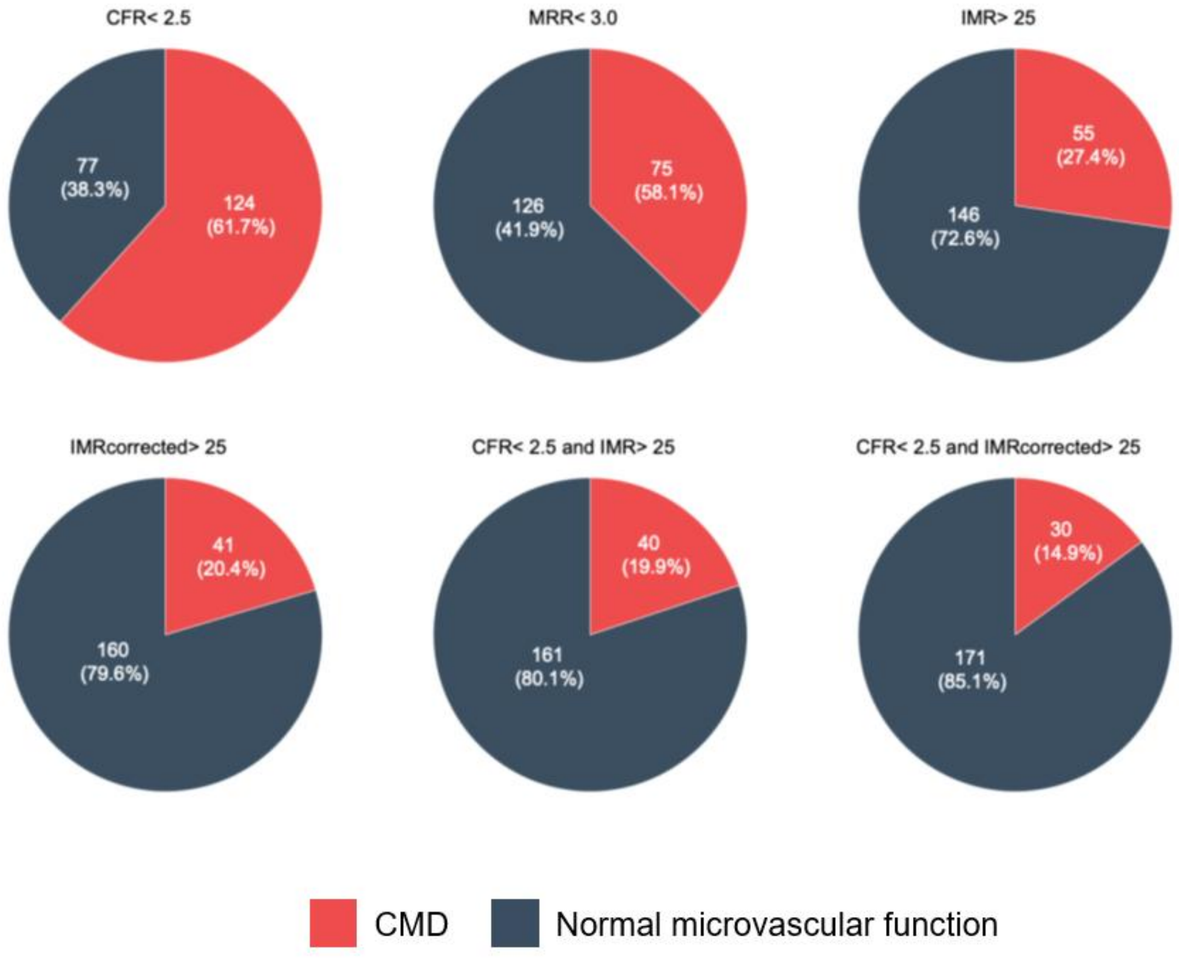

**Supplemental Figure S2. Microvascular indexes before and after PCI.**

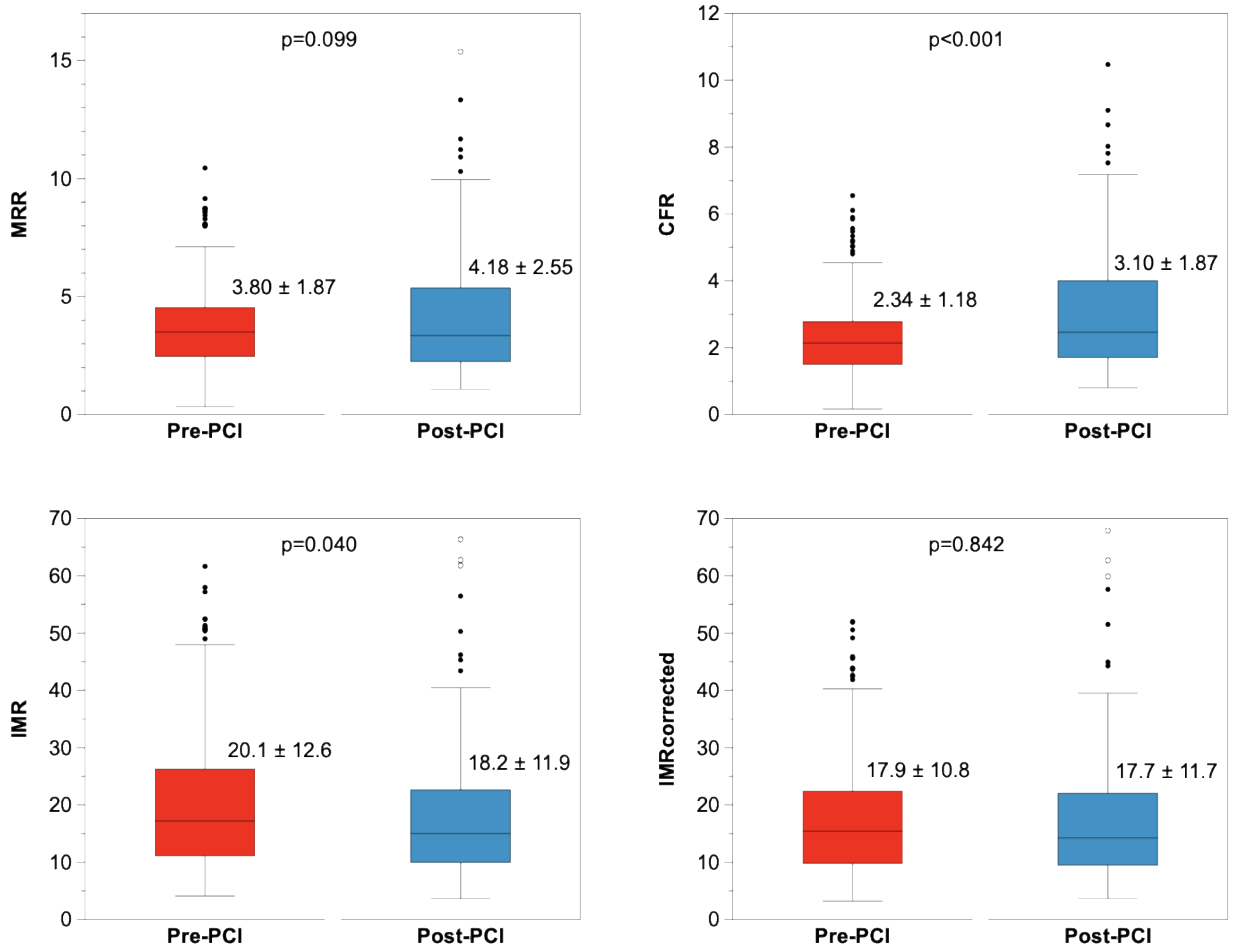

**Supplementary Tables**

**Supplementary Table S1. Comparison of clinical characteristics between patients with and without microvascular assessment.**

| Variables | All (n=993) | Microvascular assessment performed (n=242) | Microvascular assessment not performed (n=751) | p-value |
|---|---|---|---|---|
| Age, mean ± SD | 67.7 ± 10.2 | 68.2 ± 10.1 | 67.5 ± 10.2 | 0.36 |
| Male, n(%) | 757 (76.2) | 177 (73.1) | 580 (77.2) | 0.22 |
| BMI, mean ± SD | 27.0 ± 8.9 | 27.4 ± 14.4 | 26.9 ± 6.1 | 0.59 |
| Dyslipidemia, n(%) | 727 (73.2) | 178 (73.6) | 549 (73.1) | 0.95 |
| Hypertension, n(%) | 694 (69.9) | 174 (71.9) | 520 (69.2) | 0.48 |
| Diabetes, n(%) | 292 (29.4) | 83 (34.3) | 209 (27.8) | 0.06 |
| Active Tobacco, n(%) | 164 (16.5%) | 41 (16.9%) | 123 (16.4%) | 0.92 |
| Prior PCI in non-target vessel, n(%) | 277 (27.9%) | 60 (24.8%) | 217 (28.9%) | 0.26 |
| Prior PCI in target vessel, n(%) | 118 (11.9%) | 29 (12.0%) | 89 (11.9%) | 1.000 |
| Prior MI, n(%) | 197 (19.8%) | 45 (18.6%) | 152 (20.2%) | 0.642 |
| PAD, n(%) | 61 (6.1%) | 11 (4.5%) | 50 (6.7%) | 0.300 |
| Clinical presentation | | | | 0.65 |
| Non-STEMI | 57 (5.8) | 13 (5.4) | 44 (5.9) | |
| Chronic coronary Syndrome | 881 (88.9) | 215 (88.9) | 668 (88.9) | |
| Unstable angina | 53 (5.3) | 14 (5.7) | 39 (5.2) | |
| Creatinine clearance, mean±SD | 74.7 ± 27.3 | 73.8 ± 26.8 | 75.1 ± 27.5 | 0.52 |
| LVEF, mean±SD | 58.3 ± 9.5 | 58.4 ± 10.2 | 58.2 ± 9.3 | 0.78 |

*Per patient analysis*

**CMD.** Coronary Microvascular Dysfunction**; non-CMD.** non-Coronary Microvascular Dysfunction**; BMI**. Body Mass Index; **PCI**. Per-Cutaneous Intervention; **MI**. Myocardial Infarction; **PAD**. Peripheral Arterial Disease; N**on-STEMI**. Non-ST Elevation Myocardial Infarction; **LVEF**. Left Ventricular Ejection Fraction

**Supplemental Table S2: Post-PCI FFR according to epicardial pattern and CMD status**

| Epicardial pattern | CMD status | n | Post-PCI FFR mean (± SD) |
|---|---|---|---|
| Focal CAD | CMD | 44 | 0.89 ± 0.06 |
| Focal CAD | Non-CMD | 60 | 0.90 ± 0.06 |
| Diffuse CAD | CMD | 34 | 0.86 ± 0.05 |
| Diffuse CAD | Non-CMD | 67 | 0.85 ± 0.06 |

**CAD:** Coronary Artery Disease; **CMD.** Coronary Microvascular Dysfunction**; non-CMD.** non-Coronary Microvascular Dysfunction

*p*-value (CMD vs non-CMD in focal CAD): 0.23
*p*-value (CMD vs non-CMD in diffuse CAD): 0.52

**Supplemental Table S3: Comparison of baseline characteristics in patients with versus without post-PCI microvascular measurements**

| Variables | All patients (n=201) | With (n=151) | Without (n=50) | p-value |
|---|---|---|---|---|
| Age, mean ± SD | 68.5 ± 10.1 | 68.42 ± 10.13 | 68.76 ± 9.93 | 0.838 |
| Male, n (%) | 143 (71.1) | 102 (67.5) | 41 (82.0) | 0.076 |
| BMI, mean ± SD | 26.1 ± 4.0 | 26.03 ± 3.90 | 26.43 ± 4.26 | 0.535 |
| Dyslipidemia, n (%) | 147 (73.1) | 114 (75.5) | 33 (66.0) | 0.259 |
| Hypertension, n (%) | 148 (73.6) | 109 (72.2) | 39 (78.0) | 0.533 |
| Diabetes, n (%) | 65 (32.3) | 49 (32.5) | 16 (32.0) | 1.000 |
| Smoking, n(%) | 33 (16.4) | 24 (15.9) | 9 (18.0) | 0.898 |
| Prior PCI in non-target vessel, n(%) | 53 (26.4) | 44 (29.1) | 9 (18.0) | 0.245 |
| Prior PCI in target vessel, n (%) | 22 (10.9) | 16 (10.6) | 6 (12.0) | 0.818 |
| Prior MI, n (%) | 39 (19.4) | 27 (17.9) | 12 (24.0) | 0.458 |
| PAD, n (%) | 7 (3.5) | 3 (2.0) | 4 (8.0) | 0.118 |
| Clinical presentation | | | | 0.557 |
| Non-STEMI | 12 (6.0) | 10 (6.6) | 2 (4.0) | |
| Unstable angina | 10 (5.0) | 8 (5.3) | 2 (4.0) | |
| Chronic coronary Syndrome | | | | |
| Asymptomatic | 20 (10.0) | 12 (7.9) | 8 (16.0) | |
| Silent ischemia | 26 (12.9) | 19 (12.6) | 7 (14.0) | |
| Stable angina CCS I | 60 (29.9) | 47 (31.1) | 13 (26.0) | |
| Stable angina CCS II | 54 (26.9) | 40 (26.5) | 14 (28.0) | |
| Stable angina CCS III | 15 (7.5) | 13 (8.6) | 2 (4.0) | |
| Stable angina CCS IV | 4 (2.0) | 2 (1.3) | 2 (4.0) | |
| Creatinine clearance, mean ± SD | 72.5 ± 24.8 | 73.1 ± 25.4 | 70.5 ± 23.2 | 0.511 |
| LVEF, mean ± SD | 58.7 ± 10.5 | 59.3 ± 9.9 | 56.7 ± 12.0 | 0.117 |

CMD. Coronary Microvascular Dysfunction; non-CMD. non-Coronary Microvascular Dysfunction; BMI. Body Mass Index; PCI. Per-Cutaneous Intervention; MI. Myocardial Infarction; PAD. Peripheral Arterial Disease; Non-STEMI. Non-ST Elevation Myocardial Infarction; CCS. Canadian Cardiovascular Society; LVEF. Left Ventricular Ejection Fraction.

**Supplemental Table S4: Physiological predictors of residual symptoms and impaired quality of life at 12 months.**

| | Physical limitation <100 | | Angina Frequency <100 | | Quality of Life <100 | | Summary score <100 | |
|---|---|---|---|---|---|---|---|---|
| **Variables** | **Estimate (95% CI)** | **p-value** | **Estimate (95% CI)** | **p-value** | **Estimate (95% CI)** | **p-value** | **Estimate (95% CI)** | **p-value** |
| CFR <2.5 | 1.39 (0.73 to 2.70) | 0.326 | 0.98 (0.49 to 1.99) | 0.962 | 0.88 (0.49 to 1.58) | 0.661 | 1.04 (0.57 to 1.91) | 0.899 |
| MRR <3.0 | 1.76 (0.92 to 3.35) | 0.085 | 1.25 (0.62 to 2.48) | 0.528 | 1.20 (0.66 to 2.16) | 0.549 | 1.33 (0.72 to 2.48) | 0.359 |
| MRR <2.7 | 1.64 (0.84 to 3.20) | 0.144 | 1.19 (0.58 to 2.41) | 0.623 | 1.16 (0.63 to 2.15) | 0.631 | 1.28 (0.67 to 2.46) | 0.453 |
| MRR <2.1 | 1.66 (0.68 to 3.99) | 0.259 | 1.18 (0.45 to 2.88) | 0.728 | 1.21 (0.53 to 2.74) | 0.648 | 1.41 (0.59 to 3.50) | 0.446 |
| IMR >25 | 0.72 (0.35 to 1.43) | 0.353 | 1.14 (0.54 to 2.36) | 0.720 | 0.76 (0.40 to 1.43) | 0.391 | 0.83 (0.43 to 1.57) | 0.561 |
| $IMR_{corrected}$ > 25 | 0.91 (0.41 to 1.97) | 0.820 | 1.22 (0.51 to 2.75) | 0.643 | 0.93 (0.45 to 1.88) | 0.832 | 1.02 (0.49 to 2.11) | 0.958 |
| Post-PCI FFR <0.88 | 0.85 (0.46 to 1.57) | 0.598 | 1.06 (0.55 to 2.07) | 0.862 | 0.92 (0.52 to 1.62) | 0.771 | 0.96 (0.54 to 1.72) | 0.884 |
| Delta FFR < 0.15 | 1.39 (0.76 to 2.58) | 0.287 | 0.83 (0.43 to 1.61) | 0.583 | 1.16 (0.66 to 2.04) | 0.601 | 1.26 (0.71 to 2.26) | 0.437 |

**(SAQ <100; logistic models with dichotomized predictors)**

Adjusted for age and sex.

CI. Confidence interval; CFR. Coronary flow reserve; IMR. Index of microvascular resistance; MRR. Microvascular resistance reserve; FFR. Fractional Flow Reserve.

## References

1. Fearon WF, Balsam LB, Farouque HM et al. Novel index for invasively assessing the coronary microcirculation. Circulation 2003;107:3129-32.
2. Yong AS, Layland J, Fearon WF et al. Calculation of the index of microcirculatory resistance without coronary wedge pressure measurement in the presence of epicardial stenosis. JACC Cardiovasc Interv 2013;6:53-8.
3. De Bruyne B, Pijls NHJ, Gallinoro E et al. Microvascular Resistance Reserve for Assessment of Coronary Microvascular Function: JACC Technology Corner. J Am Coll Cardiol 2021;78:1541-1549.
